# Antibiotic treatment for non-specific upper respiratory tract infections and exacerbation risk in children with asthma: a target trial emulation study

**DOI:** 10.1093/aje/kwag132

**Published:** 2026-06-12

**Authors:** Carmen Cañete Ramirez, Albina Tskhay, Ana C Blanchard, Amélie Forget, Marie-Élaine Métras, Tibor Schuster, Lucie Blais, Cristina Longo

**Affiliations:** Faculté de Pharmacie, Université de Montréal, Montréal, QC, Canada; Research Centre, Centre Hospitalier Universitaire Sainte-Justine, Montréal, QC, Canada; Research Centre, Centre Hospitalier Universitaire Sainte-Justine, Montréal, QC, Canada; Department of Family Medicine, McGill University, Montréal, QC, Canada; Research Centre, Centre Hospitalier Universitaire Sainte-Justine, Montréal, QC, Canada; Faculté de Pharmacie, Université de Montréal, Montréal, QC, Canada; Faculté de Pharmacie, Université de Montréal, Montréal, QC, Canada; Research Centre, Centre Hospitalier Universitaire Sainte-Justine, Montréal, QC, Canada; Department of Family Medicine, McGill University, Montréal, QC, Canada; Faculté de Pharmacie, Université de Montréal, Montréal, QC, Canada; Faculté de Pharmacie, Université de Montréal, Montréal, QC, Canada; Research Centre, Centre Hospitalier Universitaire Sainte-Justine, Montréal, QC, Canada

**Keywords:** pediatric asthma, antibiotics, exacerbation, target trial emulation, non-specific respiratory tract infections

## Abstract

Antibiotics are often prescribed to children with asthma for non-specific upper respiratory tract infections (NS-URTIs), but their association with exacerbation risk in children with pre-existing asthma is unclear. We aimed to determine if antibiotics are associated with asthma exacerbation risk in the 6 months following NS-URTI resolution. Using Quebec administrative health and drug claims data, we emulated a series of target trials among children (0-17 years) with asthma visiting an outpatient physician for an NS-URTI. Treatment strategies were no, narrow-, or broad-spectrum antibiotics initiated within 3 days following the index visit. The primary outcome was exacerbation during the 6 months following an initial 10-day induction period, defined as oral corticosteroid use, emergency department visits, or hospitalizations for asthma. Risk ratios (RR) and differences (RDs) were estimated using inverse probability of treatment weighted log-binomial models. Among 13 646 children (24 139 person-trials), 8.8% and 11.4% received narrow- and broad-spectrum antibiotics, respectively. Compared with no antibiotics, narrow-spectrum antibiotics were associated with an RR of 1.26 (95%CI, 0.99-1.53; RD, 0.07%), while broad-spectrum antibiotics were associated with an RR of 1.28 (95%CI, 1.03-1.54; RD, 0.08%). Broad-spectrum antibiotics were associated with modestly higher exacerbation risks, although absolute RDs were small (<0.1%).

## Introduction

Asthma is the most common chronic disease in children,^1^^,^^2^ affecting 15% of children in Canada^3^ and 8.1% in the United States.^4^ Pediatric asthma has a high disease burden, ranking among the top 10 conditions for disability-adjusted life years.^2^^,^^5^ In Canada, nearly 1 in 3 emergency department (ED) visits for preschoolers are mostly due to asthma and asthma-related conditions.^6^ Moreover, asthma is also a leading cause of hospitalization among children, particularly in younger age groups.^7^ Early asthma control is important to optimize outcomes in children and improve quality of life.^8‑11^

However, most young children remain uncontrolled, with 63.3% of preschoolers having asthma or asthma-related exacerbations.^4^ Achieving asthma control during childhood is crucial to preventing airflow obstruction and lung function decline,^9^ reducing long-term risks of respiratory problems in adulthood,^10^ and potentially increasing the likelihood of remission.^11^ Thus, identifying modifiable factors contributing to exacerbations early in the disease course can potentially help avoid severe and persistent asthma in the future.^12^

One modifiable risk factor could be unnecessary antibiotic use, such as using antibiotics for indications that likely do not require them, including non-specific upper respiratory tract infections (NS-URTIs).^13^ Antibiotics can disrupt beneficial gut microorganisms, causing dysbiosis and altering gut composition and function.^14^ Broad-spectrum antibiotics reduce bacterial diversity more markedly than narrow-spectrum agents,^15^ with recovery taking up to 180 days.^16^ Macrolides appear to have a stronger disruptive effect on the gut microbiome than penicillins, which generally cause smaller shifts in taxa and resistome profiles.^17^ In addition, antibiotics may directly alter the respiratory microbiome, potentially disrupting microbial communities that help maintain airway immune homeostasis.^18^ Evidence suggests that such microbiome disruption may influence asthma control and treatment response.^19^ This may occur through the gut–lung axis, where gut dysbiosis promotes airway inflammation and may trigger exacerbations.^20^

While early-life antibiotic exposure has been associated with childhood asthma,^21‑23^ causal interpretation is limited by positivity violations, confounding by indication, and reverse causation. Many studies included children with bacterial infections for whom antibiotic use is effectively deterministic,^24^^,^^25^ while inadequate adjustment of infection indication induces confounding^23^^,^^26^^,^^27^ and early respiratory symptoms of undiagnosed asthma may further bias estimates.^23^^,^^27^ Collectively, these limitations underscore the need for study designs grounded in causal inference.

Although antibiotic use during acute asthma exacerbations has been associated with adverse outcomes in hospitalized children,^28‑30^ these findings may not generalize to outpatient settings, which accounts for 93% of antibiotic prescribing.^31^ Moreover, no studies, to our knowledge, have investigated the association between antibiotics and subsequent exacerbation in outpatient children with asthma, despite evidence that these children are more likely to receive antibiotics than those without asthma.^32^^,^^33^

This study aimed to determine, among children with asthma consulting an outpatient physician for an NS-URTI, whether broad-spectrum or narrow-spectrum antibiotics, compared with no antibiotics, is associated with exacerbation risk during the 6 months following suspected NS-URTI resolution. We hypothesized that antibiotic exposure may be associated with a higher exacerbation risk due to its potential negative influence on gut microbiota leading to higher lung inflammation, with narrower-spectrum antibiotics having less impact than broader-spectrum agents. Given the high frequency of antibiotic prescribing in children with asthma, this question has important implications for antimicrobial stewardship and prescribing decisions for indications unlikely to require antibiotics.

## Methods

### Data sources

The Quebec Asthma in Pregnancy and Infant Database (QAPID) includes validated data for asthma research in pregnant women and their children from 1990 to 2010 (the observation period), and has been described previously.^34‑37^ It includes demographic and medical service information (hospitalizations, Emergency Deparment or ED visits, and outpatient visits) with International Classification of Diseases (ICD)-9/10 diagnostic codes for mothers and children. It also includes information (type, dose, and duration) on medications dispensed to residents covered by the Quebec Public Drug Insurance Plan, which covers 43% of Quebec’s population.^38^ Data access was granted by the Institut de la Statistique de Québec (ISQ) and the study was approved by the research ethics board of the CHU Sainte Justine.

### Target trial protocol

We first specify a hypothetical target trial estimating the causal effect of antibiotic treatment strategies for NS-URTIs on asthma exacerbations in children with asthma, and then describe its emulation with QAPID data. A summary of the target trial protocol and its emulation is provided in Table S1.

#### Eligibility criteria

The target trial population consists of children aged 0**-**17 years with confirmed asthma who visit an outpatient physician for an NS-URTI. NS-URTI were chosen because prior Randomized Controlled Trials (RCTs) addressed similar indications, thereby minimizing potential positivity violations.^13^ Children are excluded if they had: (1) other chronic lung diseases (eg, cystic fibrosis or bronchopulmonary dysplasia) from birth to the index visit; (2) chronic lung infections in the year prior to the index visit; (3) an acute infection (including pneumonia) or asthma exacerbation in the month prior to the index visit; (4) an acute infection other than an NS-URTI at the index visit; (5) no public drug insurance coverage in the year before trial initiation; or (6) antibiotic use in the 6 months preceding the index visit.

#### Treatment strategies

Primary treatment strategies are: (1) no immediate antibiotic treatment; (2) immediate initiation of a narrow-spectrum antibiotic; and (3) immediate initiation of a broad-spectrum antibiotic. Broad- and narrow-spectrum antibiotics are classified using established definitions verified by the study pharmacists (Table S2).^39^ The untreated group is the reference to reflect the clinical decision point at NS-URTI presentation, where either antibiotic treatment (with a broad or narrow-spectrum antibiotic) or no treatment is selected. Secondary treatment strategies are defined by specific antibiotic class: no immediate antibiotics vs immediate initiation of: penicillins, cephalosporins, macrolides, or other antibiotics (including quinolones, lincosamides, tetracyclines, sulfonamides, or combination therapies).

#### Assignment procedures

Eligible children are randomly assigned to one of the treatment strategies at the index NS-URTI visit. Treatment assignment is ascertained within a 3-day grace period, following the index visit (T_0_), ie, the treatment ascertainment window, to reflect real-world delays between prescribing and dispensing.

#### Outcomes

The primary outcome is a moderate-to-severe asthma exacerbation occurring after a 10-day latency/induction period following the end of the treatment ascertainment window during a period of 6 months (outcome assessment window). Exacerbation is defined as at least one of the following asthma-related events: (1) an ED visit; (2) hospitalization; or (3) a short course (≤ 14 days) of oral corticosteroids. The secondary outcome is any infection-related return visit due to the potential worsening of symptoms within 10 days following the treatment ascertainment window (secondary follow-up period). This secondary outcome serves as a proximal treatment response outcome. A lack of evidence for a protective association would be consistent with the expected lack of short-term clinical benefit of antibiotics for NS-URTIs, although this remains susceptible to confounding by severity and healthcare-seeking behavior.

#### Follow-up

For the primary outcome, follow-up begins after the treatment ascertainment window and continued for 192 days. To reduce reverse causation and account for biological latency, exacerbations occurring within the first 10 days are not included in the primary analysis.^40^ This approach is consistent with randomized trials, where outcomes are restricted to events occurring after a biologically plausible induction period to improve causal interpretability; participants are followed from random assignment, but only events after this period contribute to effect estimation.^41^^,^^42^ Follow-up ends at the outcome, loss of drug insurance coverage, death, 192 days, or March 31, 2010, whichever occurrs first. For the secondary outcome, follow-up lasts 10 days after treatment ascertainment.

#### Primary causal contrasts of interest

The primary estimand is the average causal effect, defined as the difference in the 6-month risk of asthma exacerbation had all eligible children with an NS-URTI been treated with a broad-spectrum antibiotic or a narrow-spectrum antibiotic compared with if none had received antibiotics at the index visit.

For class-specific analyses, causal contrasts are defined as the difference in risk related to the primary and secondary outcomes had all children, contrary to fact, initiated a specific antibiotic class (eg, penicillins, cephalosporins, macrolides, or other) vs if none had received antibiotics at the index visit.

### Analysis plan

#### Intention-to-treat effect

The primary estimand is the intention-to-treat (ITT) effect, comparing treatment strategies initiated at the index NS-URTI visit, regardless of subsequent treatment changes. Cumulative risks under each treatment strategy are estimated and compared across strategies.

#### Subgroup analyses

Prespecified subgroup analyses assess effect modification by child characteristics and markers of asthma severity/control at baseline and care context, including age, sex, prescribing physician specialty at the index visit as well as asthma controller medication use and short-acting beta-agonist use in the year prior to the index visit.

### Emulation of the target trial using observational data

#### Eligibility criteria

Target trial eligibility criteria were operationalized using diagnostic and medication dispensing records in the QAPID. All exclusion criteria were assessed relative to the index NS-URTI visit. Chronic lung diseases were assessed from birth to the index visit, chronic lung infections - during the prior year, acute infections or asthma exacerbations - within 30 days prior to the visit, any acute infection - at the index visit not considered to be an NS-URTI, and prior antibiotic use - within the preceding 6 months. Asthma was defined using a validated Canadian algorithm requiring ≥ 1 hospitalization or ≥ 2 outpatient visits for asthma (ICD-9493; ICD-10 J45-J46) occurring between 15 and 730 days apart.^43^ This definition has reported sensitivity and specificity of 91.4% and 82.9%, respectively.^43^ Outpatient consultations for NS-URTIs were identified using ICD-9/10 codes 465.9 and J06.8 combined with a health care setting ID indicating the visit occurred in ambulatory care (000, 0X1, 6XX, 4X1, 1X3, 54×. 55×, 56×, 5X7, 340, 341, 34×, 8X5, 9X2). Children meeting eligibility criteria could contribute to multiple target trials if they experienced more than one qualifying NS-URTI during the data observation period, even if the index date of a subsequent trial occurred within the outcome window of a previous trial.

#### Treatment strategies and assignment procedures

Children were classified into treatment strategies based on antibiotic dispensing records within a 3-day treatment ascertainment window following the index visit, consistent with prior target trial emulation studies that accommodate delays between prescription and dispensing.^44^ We used inverse probability of treatment weighting (IPTW) to create a weighted population in which measured baseline covariates are balanced between intervention groups, thereby improving exchangeability.

#### Outcomes

Outcomes were ascertained using administrative claims data using diagnostic codes (ICD-9493; ICD-10 J45 for asthma exacerbation and list of infection codes in Table S3 for the secondary infection outcome), defined identically to the target trial protocol.

#### Follow-up and causal estimands

Follow-up and causal estimands were defined as in the hypothetical target trial. We estimated the observational analog of an ITT estimand, corresponding to the effect of receiving the antibiotic treatment strategy within 3-days of the index visit, regardless of subsequent treatment changes.

#### Covariates

We selected covariates that may affect the association between antibiotic treatment and asthma exacerbation based on a directed acyclic graph built from clinical knowledge and previous studies.^32^^,^^45^^,^^46^ Birth-related covariates included mode of delivery (vaginal or caesarean section), preterm birth, and low birth weight (LBW, < 2.5 kg). Covariates measured at the index visit included the child’s age, sex, rurality (urban or rural), living with a parent receiving social assistance, location of the index visit (outpatient ED vs clinic), and type of physician consulted (family doctor, pediatrician, other specialists). Asthma-related covariates measured from birth to the index visit included asthma diagnosis criteria (≥1 hospitalization vs ≥2 physician outpatient visits), year of asthma diagnosis, month of index visit, and atopy history. Asthma medication use (inhaled corticosteroids, leukotriene receptor antagonists, long-acting β2-agonists, theophylline), average weekly short-acting β2-agonist (SABA) dose, healthcare visits, and asthma exacerbations were measured during the year prior to the index visit (or from birth for children < 1 year). Maternal covariates included maternal age at the start of pregnancy, maternal asthma status, and asthma control during pregnancy as assessed using a validated index.^47^

### Analysis plan

#### Creating a sequence of target trials with the observational data

**Figure 1:**
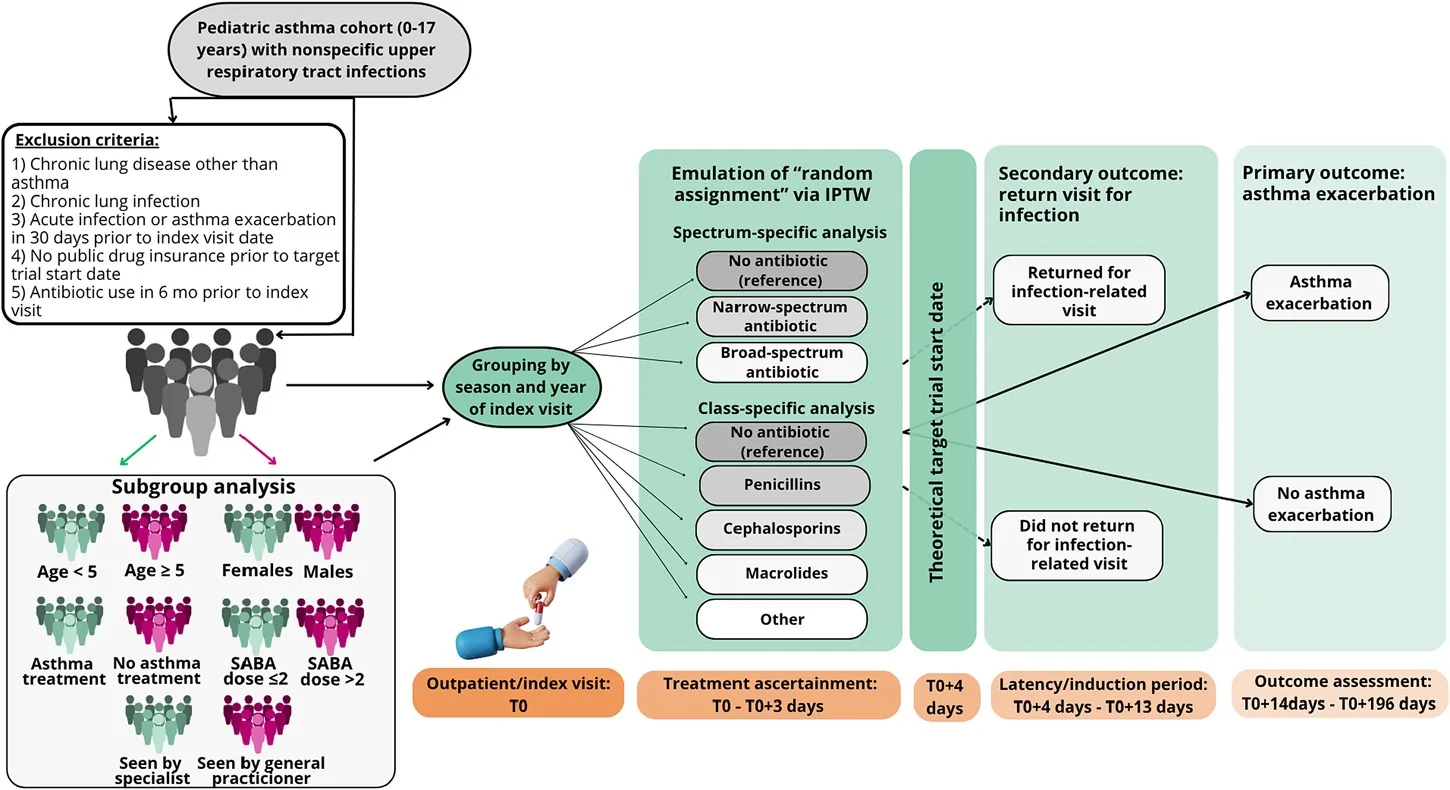
Study design schematic for a sequential target trial emulation approach comparing asthma-exacerbation risks related to narrow- and broad-spectrum antibiotic treatment vs no treatment for NS-URTIs in children with asthma. Children aged 0–17 years with asthma whopresented with a nonspecific upper respiratory tract infection (NS-URTI) were assessed for eligibility ateach potential index visit. Exclusion criteria included chronic lung disease, chronic lung infection, acuteinfection or asthma exacerbation, lack of public drug insurance, and antibiotic use. All exclusion criteriawere assessed relative to the index NS-URTI visit. Chronic lung diseases were assessed from birth to theindex visit, chronic lung infections during the prior year, acute infection or asthma exacerbation within 30 days prior to the visit, and prior antibiotic use within the preceding 6 months. Eligible visits werepaired by season and calendar year through propensity score model stratification and classified into oneof three treatment strategies based on drug claims within the 3-day treatment ascertainment window:no antibiotic (reference), narrow-spectrum antibiotic, or broad-spectrum antibiotic. For the primaryoutcome, the outcome assessment period began 14 days after the index NS-URTI visit (T0) and continuedfor 182 days. To reduce reverse causation and account for biological latency, exacerbation outcomesoccurring within the first 10 days of follow-up were not included in the primary analysis. For thesecondary outcome, follow-up began at T0+4 days until 13 days following the index visit (inclusive).Children remained eligible to re-enter subsequent trials if they again met inclusion criteria forsubsequent trials, even if the index date of a subsequent trial occurred within the outcome window of aprevious trial. Subgroup analyses were conducted by age (<5 years, ≥5 years), sex, asthma controllertherapy use, SABA use (≤2 vs. >2 doses), and type of provider (specialist vs. general practitioner).

We emulated a series of target trials occurring during the same season and calendar year, using QAPID data (Figure 1). A sequential trial approach was used to reflect the recurrent nature of acute infections in children and associated treatment decisions in clinical practice. We first assembled a base cohort of children who met eligibility criteria. We then established a theoretical time 0 (T_0_) for the target trial, defined as the moment a child with asthma meeting pre-established eligibility criteria visited an outpatient physician for an NS-URTI (the index infection visit) after their asthma diagnosis.^48^ Treatment strategies were assigned based on antibiotic dispensing 3 days after the visit. Although follow-up began after the treatment ascertainment window, events occurring during the 10-day latency/induction period were excluded from effect estimation to account for latency and avoid reverse causation.

#### Statistical analysis

We estimated IPTW via logistic regression propensity score models including all measured covariates, stratified by year and season to account for different antibiotic prescribing and exacerbation risk patterns.^48^^,^^49^ Positivity was assessed by ensuring the propensity scores were not near 0 or 1 and that stabilized weights had a mean/median near 1 without extreme values.^50^ IPT-weighted marginal structural log-binomial models^51^ were used to estimate marginal risk ratios (mRRs) and risk differences (mRDs). A clustered bootstrap was used to obtain 95% CIs accounting for repeated trials per child.^52^

#### Subgroup analyses

Prespecified subgroup analyses examined effect modification by sex, age (<5 vs ≥5 years), asthma severity proxies (controller and average SABA use in the year prior to the index visit), and prescribing physician type.

#### Sensitivity analyses

We assessed robustness by restricting to trials within 2 years of asthma diagnosis, extending follow-up to 1 year, excluding trials with exacerbations occurring during the induction period, and computing E-values for unmeasured confounding.^53^

#### Software

Analyses were performed using SAS Enterprise software, version 8.3, and R Studio version 4.3.1.

## Results

**Figure 2:**
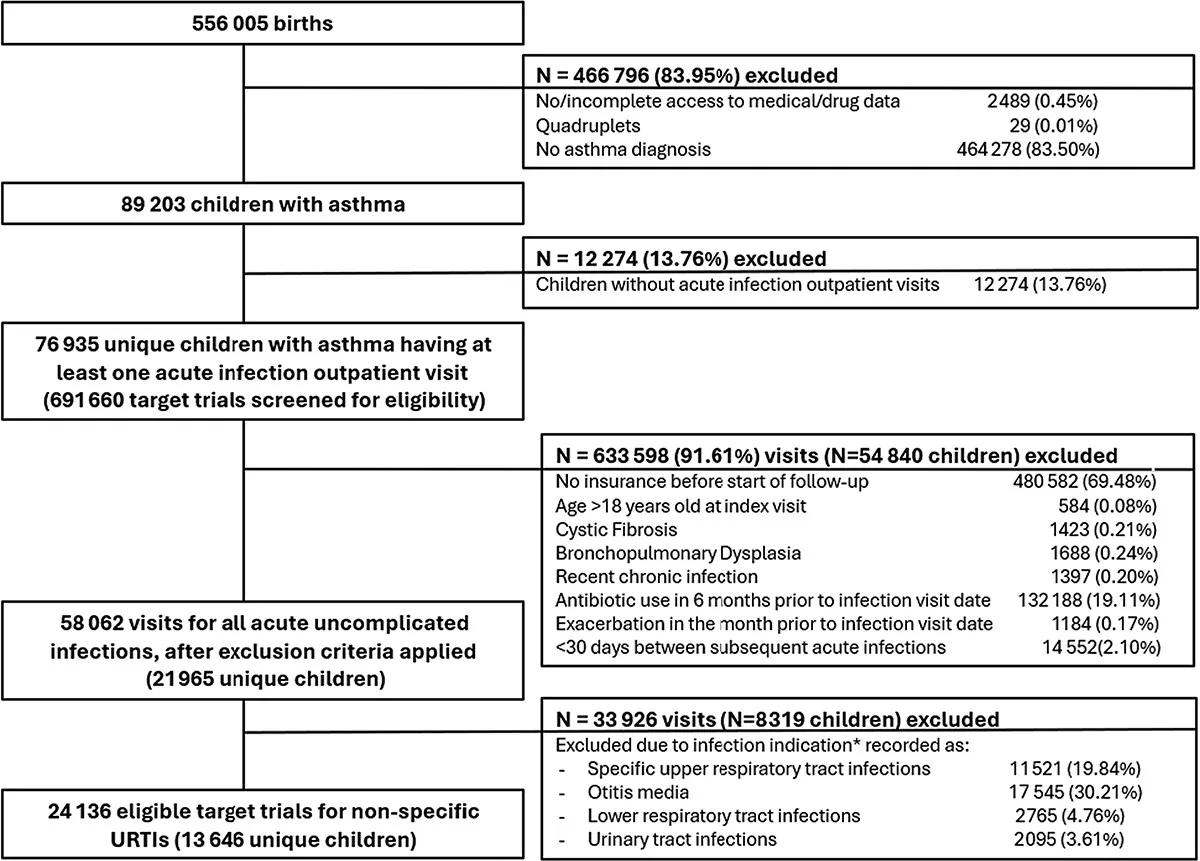
Flow chart defining the study population and included target trials. Flow diagram showing the application of exclusion criteria used to derive the analytic sample.* Specific upperrespiratory tract infections included pharyngitis, sinusitis, and tonsilitis. Lower respiratory tract infectionsincluded bronchiolitis and bronchitis. Other infections included urinary tract infections.

There were 13 646 included children with asthma contributing a total of 24 136 target trials (Figure 2). Distribution of the number of target trials contributed per child is shown in Figure S1. Among the included children, there were 659 exacerbation events over 136 262 person trial-months of follow-up (crude cumulative incidence rate of 0.48 per 100 person trial-months), with a median time-to-exacerbation of 2.5 months. Of these, 19 272 (79.8%) person-trials were not treated with antibiotics, while 2119 (8.8%) and 2745 (11.4%) were treated with narrow- and broad-spectrum antibiotics, respectively. Among those treated with antibiotics, the most frequent antibiotics dispensed across trials were penicillins (42.9%) followed by macrolides (36.9%). Compared with children treated with broad- and narrow-spectrum antibiotics, those not treated with antibiotics were consulting family doctors as well as using SABAs and other asthma controllers less often in the year prior to the index visit (Tables 1 and 2). Weighting generated balance among all measured covariates across treatment strategies (Table S4).

**Table 1:** Baseline sociodemographic, infection-related, and health care utilization characteristics of pediatric person-trials included in the evaluation of antibiotic treatment strategies for NS-URTIs and asthma exacerbation risk.

	**All trials *N* = 24 136 (100%)**	**Person-trials not treated with antibiotic *N* = 19 272 (79.8%)**	**Person-trials treated with an antibiotic**	
			**Narrow-spectrum *N* = 2119 (8.8%)**	**Broad-spectrum *N* = 2745 (11.4%)**
Males, *n* (%)	13 693 (56.7)	10 939 (56.8)	1168 (55.1)	1586 (57.8)
Children’s age in years, mean (SD)	7.2 (3.7)	7.2 (3.7)	6.9 (3.6)	7.3 (3.8)
Baby (0-1)	605 (2.5)	484 (2.5)	51 (2.4)	70 (2.6)
Toddler (>1-3)	3557 (14.7)	2855 (14.8)	323 (15.2)	379 (13.8)
Preschool (4-5)	5098 (21.1)	4022 (20.9)	484 (22.8)	592 (21.6)
Elementary school (6-12)	12 370 (51.3)	9906 (51.4)	1091 (51.5)	1373 (50.0)
Teen (>12)	2506 (10.4)	2005 (10.4)	170 (8.0)	331 (12.1)
Urban/rural, *n* (%)				
Rural	4271 (17.7)	3322 (17.2)	383 (18.1)	566 (20.6)
Urban	19 865 (82.3)	15 950 (82.8)	1736 (81.9)	2179 (79.4)
Children on social assistance, *n* (%)	11 463 (47.5)	9041 (46.9)	1151 (54.3)	1271 (46.3)
Location of consult, *n* (%)				
Outpatient clinic	21 073 (87.3)	16 936 (87.9)	1805 (85.2)	2332 (85.0)
Emergency department	3063 (12.7)	2336 (12.1)	314 (14.8)	413 (15.0)
Type of physician consulted, *n* (%)				
Family physician	19 314 (80.0)	14 990 (77.8)	1861 (87.8)	2463 (89.7)
Pediatrician	4558 (18.9)	4078 (21.2)	232 (10.9)	248 (9.0)
Other specialist	264 (1.1)	204 (1.1)	26 (1.2)	34 (1.2)
Type of antibiotic prescribed, *n* (%)				
No antibiotic	19 272 (79.8)	19 272 (100.0)	0 (0.0)	0 (0.0)
Penicillins	2087 (8.6)	0 (0.0)	1982 (93.5)	105 (3.8)
Cephalosporins	691 (2.9)	0 (0.0)	23 (1.1)	668 (24.3)
Macrolides	1795 (7.4)	0 (0.0)	94 (4.4)	1701 (62.0)
Other	291 (1.2)	0 (0.0)	68 (3.2)	223 (8.1)
Quinolones	5 (0.02)	0 (0.0)	0 (0.0)	5 (0.2)
Lincosamides	18 (0.07)	0 (0.0)	0 (0.0)	18 (0.7)
Tetracyclines	6 (0.02)	0 (0.0)	0 (0.0)	6 (0.2)
Sulfonamides	76 (0.3)	0 (0.0)	0 (0.0)	76 (2.8)
Combination of 2+ antibiotics	188 (0.8)	0 (0.0)	68 (3.2)	118 (4.3)
No. of visits by setting in the year prior to the index visit, median (IQR)				
Ambulatory care visits	4.0 (5.0)	4.0 (5.0)	4.0 (5.0)	4.0 (5.0)
ER visits	0.0 (1.0)	0.0 (1.0)	0.0 (1.0)	0.0 (1.0)
Hospitalization	0.0 (0.0)	0.0 (0.0)	0.0 (0.0)	0.0 (0.0)
No. of visits in the year prior to index visit by physician type, median (IQR)				
Family physician	3.0 (4.0)	3.0 (4.0)	3.0 (3.0)	3.0 (3.0)
Pediatrician	1.0 (3.0)	1.0 (3.0)	0.0 (2.0)	0.0 (2.0)
Specialist	0.0 (1.0)	0.0 (1.0)	0.0 (1.0)	0.0 (2.0)
No. of physician visits for acute infections from birth to the infection visit index date, *n* (%)				
0 to 4	4307 (17.8)	3396 (17.6)	451 (21.3)	460 (16.8)
5 to 7	4837 (20.0)	3857 (20.0)	457 (21.6)	523 (19.1)
8 to 10	4410 (18.3)	3531 (18.3)	378 (17.8)	501 (18.3)
11 to 15	5268 (21.8)	4235 (22.0)	444 (21.0)	589 (21.5)
16 or more	5314 (22.0)	4253 (22.1)	389 (18.4)	672 (24.5)

**Table 2:** Baseline lung function, asthma-related, and birth characteristics of pediatric person-trials included in the evaluation of antibiotic treatment strategies for NS-URTIs and asthma exacerbation risk.

	**All trials *N* = 24 136 (100%)**	**No antibiotic *N* = 19 272 (79.8%)**	**Narrow-spectrum *N* = 2119 (8.8%)**	**Broad-spectrum *N* = 2745 (11.4%)**
Age at asthma diagnosis, mean (SD)	2.3 (2.2)	2.3 (2.2)	2.1 (2.1)	2.2 (2.2)
Asthma diagnostic criteria, *n* (%)				
≥ 1 Hospitalization	4363 (18.1)	3419 (17.7)	407 (19.2)	537 (19.6)
≥ 2 Outpatient visits	19 773 (81.9)	15 853 (82.3)	1712 (80.8)	2208 (80.4)
Year of asthma diagnosis, *n* (%)				
< 1999	11 985 (49.7)	9684 (50.2)	950 (44.8)	1351 (49.2)
≥ 1999	12 151 (50.3)	9588 (49.8)	1169 (55.2)	1394 (50.8)
Season of index visit, *n* (%)				
Spring	6371 (26.4)	5050 (26.2)	576 (27.2)	745 (27.1)
Summer	3019 (12.5)	2405 (12.5)	316 (14.9)	298 (10.9)
Fall	7021 (29.1)	5764 (29.9)	551 (26.0)	706 (25.7)
Winter	7725 (32.0)	6053 (31.4)	676 (31.9)	996 (36.3)
Atopy^a^, *n* (%)	16 202 (67.1)	13 032 (67.6)	1325 (62.5)	1845 (67.2)
Asthma controller medication use in the year prior to the index visit, *n* (%)				
ICS alone	10 256 (42.5)	8113 (42.1)	877 (41.4)	1266 (46.1)
ICS-LTRA	636 (2.6)	484 (2.5)	55 (2.6)	97 (3.5)
ICS-LABA	279 (1.2)	227 (1.2)	15 (0.7)	37 (1.3)
Other controller^b^	284 (1.2)	216 (1.1)	22 (1.0)	46 (1.7)
No controller	12 681 (52.5)	10 232 (53.1)	1150 (54.3)	1299 (47.3)
Number of asthma controller claims in the year prior to the index visit, *n* (%)				
0	12 681 (52.5)	10 232 (53.1)	1150 (54.3)	1299 (47.3)
1	10 364 (42.9)	8201 (42.6)	883 (41.7)	1280 (46.6)
2	931 (3.9)	721 (3.7)	72 (3.4)	138 (5.0)
3 or more	160 (0.7)	118 (0.6)	14 (0.7)	28 (1.0)
SABA dose/week 6 months prior to index visit, *n* (%)				
0 to 2	18 859 (78.1)	15 255 (79.2)	1573 (74.2)	2031 (74.0)
>2 to <4	839 (3.5)	669 (3.5)	75 (3.5)	95 (3.5)
4 to <7	2388 (9.9)	1824 (9.5)	245 (11.6)	319 (11.6)
7 or more	2050 (8.5)	1524 (7.9)	226 (10.7)	300 (10.9)
Number of exacerbations in the year prior to the index visit, *n* (%)				
0	21 063 (87.3)	16 862 (87.5)	1840 (86.8)	2361 (86.0)
1	1592 (6.6)	1219 (6.3)	149 (7.0)	224 (8.2)
2	692 (2.9)	538 (2.8)	67 (3.2)	87 (3.2)
3 or more	789 (3.3)	653 (3.4)	63 (3.0)	73 (2.7)
Gestational age: weeks, median (IQR)	39.0 (2.0)	39.0 (2.0)	39.0 (2.0)	39.0 (2.0)
Preterm birth				
Preterm (<37 weeks)	2747 (11.4)	2175 (11.3)	258 (12.2)	314 (11.4)
Term (≥37 weeks)	21 389 (88.6)	17 097 (88.7)	1861 (87.8)	2431 (88.6)
Mode of birth				
Vaginal	18 637 (77.2)	14 884 (77.2)	1617 (76.3)	2136 (77.8)
Cesarean	2103 (8.7)	1659 (8.6)	187 (8.8)	257 (9.4)
Unknown	3396 (14.1)	2729 (14.2)	315 (14.9)	352 (12.8)
Low birth weight (<2.5 kg/5.5 lbs)	2324 (9.6)	1857 (9.6)	222 (10.5)	245 (8.9)
Maternal age at beginning of pregnancy, mean (SD)	26.0 (5.6)	26.0 (5.6)	25.8 (5.7)	26.0 (5.7)
Asthma control during pregnancy^c^				
Well controlled	1651 (6.8)	1336 (6.9)	138 (6.5)	177 (6.4)
Partially controlled	747 (3.1)	603 (3.1)	74 (3.5)	70 (2.6)
Not controlled	809 (3.4)	620 (3.2)	89 (4.2)	100 (3.6)
Could not be assessed	2367 (9.8)	1917 (9.9)	187 (8.8)	263 (9.6)
No asthma	18 562 (76.9)	14 796 (76.8)	1631 (77.0)	2135 (77.8)

Abbreviations: ICS, inhaled corticosteroids; IQR, interquartile range; LABA, long-acting beta-2 agonists; LTRA, leukotriene receptor antagonists; N/A, not applicable; SABA, short-acting beta-2 agonists.

a Atopy was defined using the following ICD-9/10 codes: 691, 692, 708, 477, 691.8, L20, L23, L50, J30.

b Other controller medications include: theophylline alone, LABA alone, leukotriene receptor antagonists alone.

c Maternal asthma defined as ≥1 hospitalization or ≥2 physician outpatient visits for asthma between 15 to 730 days apart and measured in the 2 years before child birth. Maternal asthma control could only be assessed for only mothers covered by public drug insurance and was based on a combination of SABA rescue inhaler use, exacerbations requiring emergency department visits or hospital admissions, or receipt of short-course oral corticosteroids using a Canadian database index.^47^

Compared with children not immediately treated with antibiotics, children treated with broad- or narrow-spectrum antibiotics had small absolute increases in exacerbation risk (broad-spectrum mRD, 0.08%; 95%CI, 0.01%**-**0.14%; narrow-spectrum mRD, 0.07%; 95%CI, 0.00%**-**0.14%), corresponding to relative risks of 1.28 (95%CI, 1.03-1.54) and 1.26 (95%CI, 0.99**-**1.53), respectively. However, the CIs generated for narrow-spectrum antibiotics were also consistent with no effect. In class specific analyses, penicillins were not associated with exacerbation risk (mRR, 1.01; 95%CI, 0.83-1.22; mRD, 0.01%, 95%CI, -0.04% to 0.06%), whereas cephalosporins, macrolides, and the other category (dominated by combination antibiotics and others) tended to increase average risk, on average, relative to no antibiotics, with mRRs of 1.16 (95%CI, 0.78-1.56), 1.24 (95%CI, 0.92-1.55), and 1.69 (95%CI, 0.86-2.50), respectively (Table 3). Nevertheless, CIs were also consistent with no increase in risk. Moreover, treatment with narrow or broad-spectrum antibiotics did not reduce the risk of infection-related return visits in the primary analyses; however, class-specific analyses showed that macrolides may be associated with a lower risk (mRR, 0.87; 95%CI, 0.75-1.01) (Table 4).

**Table 3:** RRs and RDs for exacerbation associated with antibiotic treatment strategies in children with asthma consulting for NS-URTIs.

	**No. of first exacerbations/no. of person-trial months**	**Crude incidence rate (95% CI) per 100 person-trial months**	**Crude RRs** ^**a**^ **(95% CI)**	**Marginal RRs** ^**b**^ **(95% CI)**	**Crude RD** ^**a**^ **(95%CI)**	**Marginal RD** ^**b**^ **(95% CI)**
All	659/136261.7	0.48 (0.45; 0.52)	N/A	N/A	N/A	N/A
No antibiotic^c^	464/100941.9	0.46 (0.42; 0.50)	Ref	Ref	Ref	Ref
Spectrum-specific effects						
Narrow	77/14547.7	0.53 (0.41; 0.65)	1.15 (0.90; 1.47)	1.26 (0.99; 1.53)	0.07% (−0.06%; 0.20%)	0.07% (0.00%; 0.14%)
Broad	118/20772.1	0.57 (0.47; 0.67)	1.24 (1.01; 1.51)	1.28 (1.03; 1.54)	0.11% (0.00%; 0.22%)	0.08% (0.01%; 0.14%)
Class effects						
Penicillins	55/11621.1	0.47 (0.36; 0.62)	1.03 (0.78; 1.35)	1.01 (0.83; 1.22)	0.01% (−0.08%; 0.09%)	0.01% (-0.04%; 0.06%)
Cephalosporins	21/3843.167	0.55 (0.34; 0.84)	1.20 (0.77; 1.84)	1.16 (0.78; 1.56)	0.09% (−0.05%; 0.22%)	0.05% (-0.04%;0.12%)
Macrolides	63/9970.4	0.63 (0.49; 0.81)	1.37 (1.04; 1.79)	1.24 (0.92; 1.55)	0.17% (0.06%; 0.28%)	0.06% (0.02%; 0.12%)
Other^d^	13/1606.3	0.81 (0.43; 1.38)	1.75 (1.00; 3.05)	1.69 (0.86; 2.50)	0.35% (0.00%; 0.69%)	0.17% (-0.01%; 0.32%)

Abbreviations: NS-URTI, non-specific upper respiratory tract infection; RD, risk difference; RR, risk ratios; SABA, short-acting beta-2 agonist.

a Crude effects are unadjusted for confounders.

b Marginal effects are adjusted for confounders via inverse probability of treatment weighting. Confounders included in the propensity score model used to estimate weights include: sex, age, rurality, social assistance, location of index visit, type of physician at index visit, asthma diagnosis criteria, year of asthma diagnosis, month of the index visit, atopy, asthma controller medication, average weekly dose of short-acting beta-2 agonist, number of asthma exacerbation in the previous year, number of health care visits for any cause at outpatient clinic in the previous year, low birth weight, preterm birth, mode of delivery, maternal age at the beginning of pregnancy, maternal asthma status, and asthma control during pregnancy.

c Reference category.

d The other category includes combination of 2 or more antibiotics from the list: Amoxicillin, Benzathine, Clarithromycin, Clavunac, Penicillin V, as well as more rare class antibiotics: quinolones, lincosamides, tetracyclines, and sulfonamides.

**Table 4:** RRs and RDs for infection-related return visits associated with antibiotic treatment strategies in children with asthma consulting for NS-URTIs.

	**No. of infection-related return visits/no. of person-trial months**	**Crude incidence rate (95% CI) per 100 person-trial months**	**Crude RRs** ^**a**^ **(95% CI)**	**Marginal RRs** ^**b**^ **(95% CI)**	**Crude RD** ^**a**^ **(95%CI)**	**Marginal RD** ^**b**^ **(95% CI)**
All	1173/134292.1	0.87 (0.82; 0.92)	N/A	N/A	N/A	N/A
No antibiotic^c^	948/107251.1	0.88 (0.83; 0.94)	ref	ref	ref	ref
Spectrum-specific effects						
Narrow	96/11761.9	0.82 (0.66; 1.00)	0.92 (0.75; 1.14)	0.92 (0.75; 1.09)	−0.07% (−0.24%; 0.11%)	-0.04% (-0.11%; 0.04%)
Broad	129/15279.1	0.84 (0.70; 1.00)	0.96 (0.80; 1.15)	1.01 (0.87; 1.16)	−0.04% (−0.20%; 0.12%)	0.01% (-0.07%; 0.08%)
Class effects						
Penicillins	99/11621.1	0.85 (0.69; 1.04)	0.97 (0.79; 1.18)	1.00 (0.83; 1.16)	−0.03% (−0.11%; 0.06%)	-0.01% (-0.09%; 0.06%)
Cephalosporins	38/3843.167	0.99 (0.70; 1.36)	1.13 (0.84; 1.49)	1.10 (0.87; 1.37)	0.11% (−0.07%; 0.28%)	0.05% (-0.10%; 0.19%)
Macrolides	73/9970.4	0.73 (0.57; 0.92)	0.83 (0.67; 1.03)	0.87 (0.75; 1.01)	−0.15% (−0.27%;−0.02%)	-0.06% (-0.12%; 0.0%)
Other^d^	15/1606.3	0.93 (0.52; 1.54)	1.06 (0.68; 1.63)	1.13 (0.82; 1.44)	0.05% (−0.17%; 0.27%)	0.07% (-0.20%; 0.26%)

Abbreviations: NS-URTI, non-specific upper respiratory tract infection; RD, risk difference; RR, risk ratios; SABA, short-acting beta-2 agonist.

a Crude effects are unadjusted for confounders.

b Marginal effects are adjusted for confounders via inverse probability of treatment weighting. Confounders included in the propensity score model used to estimate weights include: sex, age, rurality, social assistance, location of index visit, type of physician at index visit, asthma diagnosis criteria, year of asthma diagnosis, month of the index visit, atopy, asthma controller medication, average weekly dose of SABA, number of asthma exacerbation in the previous year, number of health care visits for any cause at outpatient clinic in the previous year, low birth weight, preterm birth, mode of delivery, maternal age at the beginning of pregnancy, maternal asthma status, and asthma control during pregnancy.

c Reference category.

d The other category includes combination of 2 or more antibiotics from the list: Amoxicillin, Benzathine, Clarithromycin, Clavunac, Penicillin V, as well as more rare class antibiotics: quinolones, lincosamides, tetracyclines, and sulfonamides.

**Figure 3:**
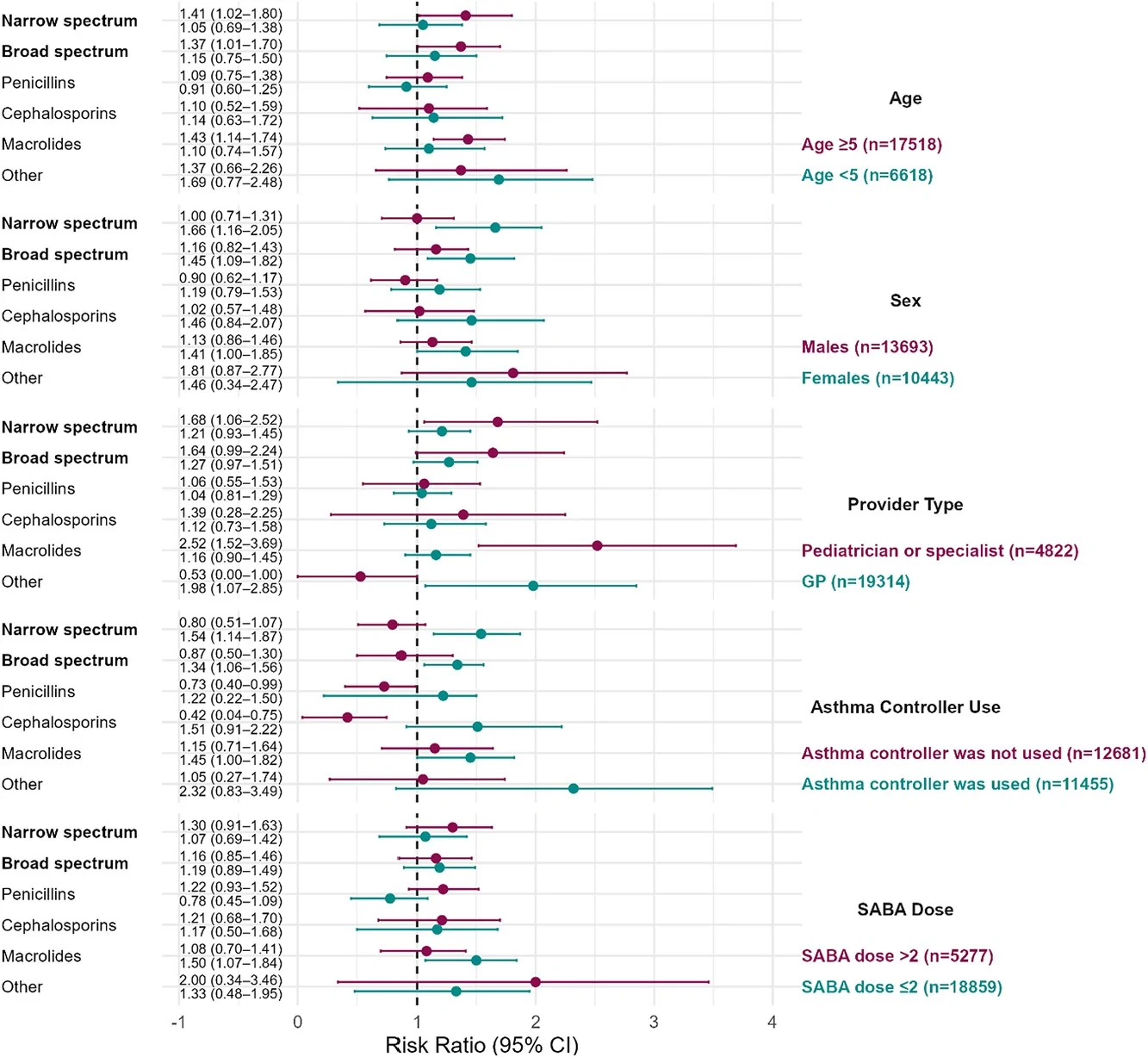
Antibiotic exposure and asthma exacerbation risk: subgroup effects in children with NS-URTI. The graph displays the Marginal Risk Ratio (mRR) with a 95% Confidence Interval (CI) comparing children exposed to spectrum-specific and class-specific antibioticsversus those unexposed, following a non-specific acute upper respiratory infection. The results arestratified into several subgroups, including age, sex, the type of doctor seen, use of asthma controllermedications, and average weekly dose of short-acting beta-2 agonist (SABA) use in the year before theindex visit.

In subgroup analyses, the modest increases in exacerbation risk associated with broad- and narrow-spectrum antibiotics appeared to be more pronounced among children aged 5 years and older. Associations also tended to be higher in girls, children seen by a specialist, and those who received asthma treatment in the year prior to the index visit (Figure 3). For the infection-related return visit outcome, broad-spectrum antibiotic associations were higher among girls, children seen by a specialist, and those using asthma controllers in the year prior to the index visit (Figure S2).

All sensitivity analyses yielded estimates comparable to those of the main analysis (Tables S5-S7). E-values suggested that lower CIs and average observed associations would require weak and moderate unmeasured confounding, respectively, to be fully explained away (Table S8).

## Discussion

In this target trial emulation study within a large cohort of children with asthma consulting an outpatient physician for an NS-URTI, we observed modestly higher risks of subsequent asthma exacerbation among children receiving broad- and narrow-spectrum antibiotics compared with no treatment. However, the corresponding absolute RDs were small (<0.1%), and the CIs for narrow-spectrum antibiotics were consistent with both a small increase in risk and no association. Moreover, antibiotic treatment was not associated with a reduction in infection-related return visits. In class-specific secondary analyses, penicillins had little to no association with exacerbation risk on average, whereas macrolides, cephalosporins, and other antibiotics (primarily combination antibiotics) tended to increase average exacerbation risk relative to no treatment. However, these estimates were imprecise, with wide 95% CIs that included the null and overlapped across antibiotic classes; accordingly, these class-specific findings should be interpreted cautiously and considered hypothesis-generating only.

The observed associations between broad- and narrow-spectrum antibiotic use and subsequent asthma exacerbations are broadly consistent with previous studies linking antibiotic use during asthma exacerbations to adverse asthma outcomes, although the magnitude of association observed in our study was smaller and those studies were conducted primarily among hospitalized patients.^28‑30^ Evidence from population-based and twin cohorts also suggests that early-life exposure to both broad- and narrow-spectrum antibiotics is associated with increased asthma risk, although these findings may be influenced by confounding by indication or reverse causation.^27^^,^^54^ In contrast, our study focused on children with pre-existing asthma, NS-URTIs as an indication to reduce positivity violations and confounding by indication, and used a target trial emulation framework with IPTW to reduce self-inflicted biases and strengthen causal interpretation.^55^

While our findings suggest small associations, antibiotics may, in theory, increase exacerbation risk through microbiome-mediated mechanisms. Antibiotics can disrupt gut microbial diversity and reduce production of immunomodulatory metabolites such as short-chain fatty acids, impairing regulatory T-cell function and promoting pro-allergic Th2 dominant immune responses.^56^ Antibiotics with broader vs narrower activity have been shown to have a greater negative effect on the intestinal microbiota,^15^ potentially leading to greater inflammation and immune response in the lungs via the gut–lung axis. Specifically, macrolides and cephalosporins have been shown to have the most pronounced and long-lasting effects, with microbial disruption persisting for months to years, and have been linked to a higher risk of wheeze and asthma later in childhood.^57^^,^^58^ These effects are twice as long-lasting when compared with penicillins.^58^ Taken together, our class-specific findings are broadly consistent with existing evidence suggesting that antibiotics with greater microbiome-disrupting potential may be associated with higher exacerbation risk. However, given the imprecision of our class-specific estimates and the substantial overlap in CIs across antibiotic classes, these findings should be considered exploratory and hypothesis-generating. In addition, a modest risk reduction for infection-related return visits was also observed for macrolides. One possible explanation for this finding is azithromycin’s immunomodulatory and anti-inflammatory properties that may confer modest symptomatic benefit in viral respiratory infections^59^^,^^60^; however, the clinical relevance of this effect in NS-URTIs remains uncertain.

We also found that both broad- and narrow-spectrum antibiotic effects on exacerbation appeared to be more pronounced among children aged 5 years and older, girls, children seen by a specialist at the index visit, and those using asthma controller therapy in the year prior to the index visit. The lower effect observed in children under 5 years old may be due to the nature of preschool asthma, which is frequently transient and remits before school age.^61^ As a result, a higher proportion of remitting children in the preschool subanalysis may have diluted effect estimates when compared with older children, who often have persistent asthma. Age-related differences may also reflect variation in the underlying etiology of respiratory symptoms, with viral infections predominating in younger children^62^ and potentially more heterogeneous inflammatory contributions in older children, ^63^as well as developmental differences in airway and gut microbiome composition that may influence immune responses and susceptibility to exacerbations.^64^ The stronger antibiotic–exacerbation associations observed in girls may reflect sex-specific differences in immune development and different patterns of antibiotic-induced gut metabolome disruption, which can disproportionately impair the development of immune tolerance.^65^ This is particularly relevant since females have been shown to be more susceptible to dysregulated immune responses, including higher rates of allergic and autoimmune conditions.^66^^,^^67^ Lastly, the higher antibiotic-exacerbation risks observed in children seen by specialists at the index visit and in those using asthma controller medications may reflect differences in underlying asthma persistence, severity, healthcare utilization, and healthcare-seeking behaviors. Although residual differences between patient groups may partly explain these findings, they may also align with recent findings that patients with allergic asthma may be more sensitive to microbiome disruptions leading to higher airway inflammation, which can affect asthma control.^68^

Strengths of this study include the use of a target trial emulation framework in a large, well-validated administrative cohort of children with asthma, with extensive confounder adjustment.

Nevertheless, this study also has limitations. First, residual confounding by infection severity or etiology may persist despite adjustment for proxies including healthcare utilization, number of prior infection visits, visit setting, and physician type, which achieved good covariate balance across treatment groups. Because the specific viral pathogen responsible for the NS-URTI could not be identified, differential distributions of more severe viral infections between treatment groups cannot be excluded, although a 10-day delayed risk window was used to reduce the likelihood that outcomes reflected the severity of the presenting infection itself. In addition to residual confounding by indication, confounding from other unmeasured factors such as obesity and race/ethnicity may also remain,^69‑72^ with E-value analyses suggesting that weak unmeasured confounding could attenuate the observed effects. Second, non-differential misclassification of the outcome may have occurred due to reliance on administrative codes to define exacerbations. Third, positivity violations are possible if some bacterial infections were incorrectly coded as NS-URTIs and were more likely to receive antibiotics, but propensity score overlap was excellent after restricting to children without recent infection or antibiotic exposure. Fourth, unmeasured physician-level factors may have influenced antibiotic prescribing for NS-URTIs. Although prescriber preference could theoretically serve as an instrumental variable, the large number of prescribers, each contributing relatively few patients, limits the feasibility of defining a strong, stable instrument. Moreover, this approach would require strong assumptions that may not be testable in this context. We attempted to mitigate potential confounding by adjusting for and stratifying by physician type at the index visit (general practitioner, pediatrician, or other specialist), both to reduce confounding and to explore potential effect modification. Fifth, limited sample sizes within antibiotic class strata led to imprecise class-specific effects with wide CIs, with some subgroup analyses exhibiting instability. Finally, the ITT framework does not account for antibiotic use during follow-up, which may contribute to cumulative exposure but cannot be easily addressed without introducing time-varying bias.

In this large target trial emulation study of children with asthma consulting an outpatient physician for an NS-URTI, broad- and narrow-spectrum antibiotic treatments were associated with modestly higher exacerbation risks than no antibiotic treatment, although the absolute RDs were small (<0.1%) and CIs for narrow-spectrum antibiotics were also consistent with no association. Since antibiotic prescribing rates are highly prevalent in children with vs without asthma,^32^ these findings further reinforce current antimicrobial stewardship principles, where antibiotics should be prescribed only when there is a strong clinical suspicion of bacterial infection. Although the observed absolute increase in exacerbation risk associated with antibiotic treatment for NS-URTIs was small, this potential adverse effect may be considered alongside other risks and benefits when making treatment decisions in children with asthma. Because residual confounding by indication or infection severity cannot be completely excluded, these findings should be interpreted cautiously and warrant confirmation in other populations and data sources.

## Supplementary Material

Web_Material_kwag132

## Data Availability

The data underlying this article were provided by the Québec Asthma and Pregnancy Infant Database (QAPID) under data-sharing agreements and are not publicly available. The authors do not have permission to share or redistribute the data due to privacy and confidentiality restrictions imposed by the Institut de la statistique du Québec.

## Notes

Author Contributions: Carmen Cañete Ramirez and Albina Tskhay contributed equally as first co-authors. Cristina Longo had full access to all the data in the study and take responsibility for the integrity of the data and the accuracy of the data analysis. Carmen Cañete Ramirez contributed to the design of the study, performed the data analysis, interpreted the results, wrote and revised the manuscript. Albina Tskhay performed the data analysis, interpreted the results, and wrote and revised the manuscript. Cristina Longo conceived and designed the study, acquired funding and the data, supervised Carmen Cañete Ramirez and Albina Tskhay, contributed to the analysis and interpretation of the results, as well as revised and approved the manuscript. Lucie Blais helped to acquire the data, contributed to methods, interpretation of results, and revised the manuscript. Amélie Forget cleaned the data, created the cohorts, contributed to the interpretation of the results, and revised and approved the manuscript. Tibor Schuster, Marie-Élaine Métras, and Ana Maria Blanchard contributed to methods, interpretation of results, and revised the manuscript.
