# Supplementary material for: Antibiotic treatment for non-specific upper respiratory tract infections and exacerbation risk in children with asthma: a target trial emulation study
Source: Am J Epidemiol. 2026 Jun 12;195(9):2334–46. doi: 10.1093/aje/kwag132 (PMC13537853; doi:10.1093/aje/kwag132)
Supplement: Supplementary file 1 [file web_material_kwag132.docx]

**Supplementary Material**

**Antibiotic treatment for non-specific upper respiratory tract infections and exacerbation risk in children with asthma: A target trial emulation study**

Carmen Cañete Ramirez, Albina Tskhay, Ana C. Blanchard, Amélie Forget, Marie-Élaine Métras, Tibor Schuster, Lucie Blais, Cristina Longo

Table of Contents

[Table S1. Ideal Pragmatic Trial and Emulated Trial Protocols for the Pediatric Asthma Project	2](#_Toc231827312)

[Table S2. Antibiotics used to define antibiotic exposure classified by antibiotic group and spectrum	5](#_Toc231827313)

[Table S3. Diagnosis Codes Used to Define Acute Infection Diagnosis	6](#_Toc231827314)

[Figure S1. Distribution of the number of target trials contributed per child.	7](#_Toc231827315)

[Table S4. Study Population Characteristics for Nonspecific URTIs After Weighting	8](#_Toc231827316)

[Figure S2. Antibiotic Treatment and Return for Infection Risk: Subgroup Effects in Children with Nonspecific Upper Respiratory Tract Infections	11](#_Toc231827317)

[Table S5. Sensitivity Analysis: Crude and Marginal Risk Differences and Ratios for Exacerbation over a 1-year Follow-Up Period Associated with Antibiotic Treatment Strategies in Children with Asthma Consulting for Nonspecific URTIs	12](#_Toc231827318)

[Table S6. Sensitivity Analysis: Crude and Marginal Risk Differences and Ratios for Exacerbation associated with Antibiotic Treatment Strategies in Children with Newly Diagnosed Asthma consulting for Nonspecific URTIs	13](#_Toc231827319)

[Table S7. Sensitivity Analysis: Crude and Marginal Risk Differences and Ratios for Exacerbation Associated with Antibiotic Exposure Excluding Children with an Exacerbation Between the Index Visit and the Start of Follow-up	14](#_Toc231827320)

[Table S8. E-Value Calculation for Overall Risk Ratios of Exacerbation for Each Antibiotic Treatment Strategy	15](#_Toc231827321)

## **Table S1. Ideal Pragmatic Trial and Emulated Trial Protocols for the Pediatric Asthma Project**

| **Protocol Element** | **Ideal Target Trial** | **Target Trial Emulation Using Health Administrative Data in Quebec** |
| --- | --- | --- |
| **Eligibility Criteria** | - Children aged 0-17 years old with asthma confirmed by a physician - Must visit an outpatient physician for a non-specific upper respiratory tract infection after an asthma diagnosis (index visit) - No recent use of antibiotics in the last 6 months - No chronic lung infections in the last year - No recent history of an acute infection in the last month - No documented history of other chronic lung disease from birth to the index visit - No wheezing or worsening of asthma symptoms in the previous week or at the visit | - Asthma diagnostic algorithm that is validated using Canadian data - Infection ICD-9/10 codes in consultation with a pediatric infectious disease specialist - List of antibiotic drug identification numbers (DINs) in consultation with a pharmacist - List of ICD-9/10 codes for other chronic lung diseases and infections in children from birth or one year prior to the index visit date - Minimum lookback of 1 year (must be insured by public drug insurance for at least 1 year lookback to the index visit) - Move trial start date to 14 days after visit to avoid reverse causation from exacerbations potentially present at initial visit, exclude patients with an exacerbation occurring within the month prior to index visit. |
| **Treatment Strategies (TS)** | Primary Definition:  TS2: Immediate treatment with a broad-spectrum antibiotic  TS1: Immediate treatment with a narrow-spectrum antibiotic  TS0: No immediate antibiotic treatment  Secondary Definition:  TS4: Immediate treatment with 2 or more antibiotic classes  TS3: Immediate treatment with a macrolide antibiotic class  TS2: Immediate treatment with a cephalosporin antibiotic class  TS1: Immediate treatment with a penicillin class antibiotic  TS0: No immediate antibiotic treatment | Same definitions – however, antibiotic treatment strategies ascertained within 3 days of visit to allow for sufficient time to claim antibiotics at a community pharmacy. |
| **Assignment** | Participants are randomly assigned to a strategy and are aware of their assigned strategy | Emulated using season-specific inverse probability of treatment weighting including confounders and covariates measured in the year prior to the index visit. |
| **Follow-Up** | For each eligible child, follow-up begins at assignment (baseline or time zero) and continues until development of the outcome of interest, lost-to-follow-up, or administrative end of follow-up (6 months after baseline), whichever occurs first. | Follow-up begins 4 days after the index visit (first day after treatment assignment window). For the primary outcome, events during the induction period, i.e. within the first 10 days of follow-up, were excluded to account for latency and reduce reverse causation. |
| **Outcomes** | Primary Outcome:  Asthma exacerbation occurring after the 10-day latency period as defined by an oral corticosteroid burst, emergency department visit, or hospitalization for asthma  Secondary Outcome:  An infection-related return visit (see Table S2 for list of infection codes) from the index visit + 4 days until 14 days following the index visit. | Same, including DIN codes for oral corticosteroids, ICD-9/10 codes for asthma-related emergency or hospital admissions. |
| **Causal Contrast** | Intention-to-Treat (ITT) effect | Observational analogue of the ITT effect |
| **Statistical Analysis Plan** | Marginal risk differences and risk ratios computed via IPTW weighted log-binomial models and/or modified Poisson models in the event of convergence issues with the log-binomial models. Standard errors computed with clustered bootstrap to account for a child contributing multiple trials to the analysis | Same. |

## **Table S2. Antibiotics used to define antibiotic exposure classified by antibiotic group and spectrum**

| **Group** | **Spectrum** | **Antibiotic** |
| --- | --- | --- |
| Cephalosporins | Narrow | Cephalexin |
|  | Broad | Cefaclor, Cefixime, Cefprozil, Cefuroxime |
| Macrolides | Narrow | Erythromycin |
|  | Broad | Azithromycin, Clarithromycin, Telithromycin |
| Penicillins | Narrow | Amoxicillin, Cloxacillin, Penicillin V, Penicillin V-Benzathine, Pivampicillin |
|  | Broad | Amoxicillin/ Clavulanic |
| Other Antimicrobials | Broad | Ciprofloxacin, Clindamycin, Levofloxacin, Minocycline, Sulfamethoxazole, Trimethoprim/ Sulfamethoxazole |

## **Table S3. Diagnosis Codes Used to Define Acute Infection Diagnosis**

| **Condition** | **Code ICD-9** | **Code ICD-10** |
| --- | --- | --- |
| **Acute sinusitis** | **461** | **J01** |
| **Otitis media** | **381, 382, 381.01, 381.00, 382.0, 382.4, 382.9** | **H65, H66, H65.0, H65.1, H65.9, H66.0, H66.4, H66.9** |
| **Pharyngitis** | **462** | **J02** |
| **Tonsillitis** | **463** | **J03** |
| **Common cold** | **460** | **J00** |
| **Non-specific upper respiratory tract infection** | **465** | **J06** |
| **Bronchitis** | **466.0** | **J20** |
| **Bronchiolitis** | **466.1** | **J21** |
| **Urinary tract infections** | **595.0, 595, 599.0** | **N30.0, N30, N39.0** |
| **Cellulitis and Abscess** | **682.0, 682.1, 682.2, 682.5, 682.3, 682.8, 682.9, 681, 682, 681.0, 681.1, 682.3, 682.0, 682.2, 682.8, 682.9** | **L02.0, L02.1, L02.2, L02.3, L02.4, L02.8, L02.9, L03.0, L03.1, L03.2, L03.3, L03.8, L03.9** |

## **Figure S1. Distribution of the number of target trials contributed per child.**

Most children contributed to a small number of trials, with progressively fewer children contributing to larger numbers of trials. The mean number of trials per child was 1.77 (SD 1.22).

## **Table S4. Study Population Characteristics for Nonspecific URTIs After Weighting**

|  | **All Trials**  **23,444.74**  **(100%)** | **Person-Trials Not Treated with Antibiotic**  **19,163.47**  **(81.6%)** | **Person-Trials Treated with an Antibiotic** | | **Standardized mean difference (SMD)** |
| --- | --- | --- | --- | --- | --- |
|  |  |  | **Broad Spectrum**  **2,474.97**  **(10.6%)** | **Narrow Spectrum**  **1,806.31**  **(7.8%)** |  |
| **Child Sociodemographic Variables** |  |  |  |  |  |
| **Males** | 13,270.0 (56.6) | 10,873.0 (56.7) | 1,434.5 (58.0) | 962.5 (53.3) | 0.063 |
| **Age of child, mean (SD)** | 7.25 (3.74) | 7.26 (3.75) | 7.29 (3.78) | 7.10 (3.6) | 0.034 |
| Baby (0-1 year) | 567.1 (2.4) | 466.3 (2.4) | 60.8 (2.5) | 40.1 (2.2) |  |
| Toddler (> 1-3 years) | 3,359.5 (14.3) | 2,775.7 (14.5) | 338.3 (13.7) | 245.4 (13.6) | 0.061 |
| Preschool (> 3-5 years) | 4,930.0 (21.0) | 3,991.5 (20.8) | 528.9 (21.4) | 409.6 (22.7) |  |
| Gradeschool (> 5-12 years) | 12,128.4 (51.7) | 9,918.2 (51.8) | 1,264.4 (51.1) | 945.7 (52.4) |  |
| Teen (> 12 years) | 2,459.8 (10.5) | 2,011.8 (10.5) | 282.5 (11.4) | 165.5 (9.2) |  |
| **Urban/rural** |  |  |  |  |  |
| Rural | 4,144.3 (17.7) | 3,367.0 (17.6) | 450.6 (18.2) | 326.7 (18.1) | 0.011 |
| Urban | 19,300.4 (82.3) | 15,796.47 (82.4) | 2,024.37 (81.8) | 1,479.61 (81.9) |  |
| **Infection Characteristics** |  |  |  |  |  |
| **Location of Consult** |  |  |  |  |  |
| Outpatient Clinic | 20,470.6 (87.3) | 16,847.1 (87.9) | 2,102.9 (85.0) | 1,520.4 (84.2) |  |
| Emergency Department | 2,974.1 (12.7) | 2,316.4 (12.1) | 372.1 (15.0) | 285.6 (15.8) | 0.072 |
| **Type of Physician consulted** |  |  |  |  |  |
| Pediatrician | 4,283.6 (18.3) | 3,640.5 (19.0) | 372.5 (15.1) | 270.6 (15.0) | 0.082 |
| Family physician | 18,925. 6 (80.7) | 15,319.0 (79.9) | 2,082.5 (84.1) | 1,524.0 (84.4) |  |
| Other Specialist | 235.6 (1.0) | 203.9 (1.1) | 19.9 (0.8) | 11.7 (0.6) |  |
| **Proxies of severity** |  |  |  |  |  |
| **Number of physician visits for acute infections from birth to T0, median (IQR)** | 10.0 (9.0) | 10.0 (9.0) | 10.0 (9.0) | 10.0 (9.0) | 0.043 |
| 0 to 4 | 4,110.8 (17.5) | 3,341.2 (17.4) | 419.2 (16.9) | 350.5 (19.4) | 0.052 |
| 5 to 7 | 4,679.7 (20.0) | 3,819.2 (19.9) | 502.5 (20.3) | 358.0 (19.8) |  |
| 8 to 10 | 4,279.3 (18.3) | 3,507.6 (18.3) | 458.7 (18.5) | 313.1 (17.3) |  |
| 11 to 15 | 5,169.5 (22.0) | 4,225.0 (22.0) | 536.7 (21.7) | 407.8 (22.6) |  |
| 16 or more | 5,205.3 (22.2) | 4,270.5 (22.3) | 557.9 (22.5) | 376.9 (20.9) |  |
| **Asthma-Related Characteristics** |  |  |  |  |  |
| **Age at asthma diagnosis, mean (SD)** | 2.27 (2.23) | 2.29 (2.24) | 2.22 (2.19) | 2.20 (2.14) | 0.028 |
| **Asthma diagnostic criteria** |  |  |  |  |  |
| ≥1 Hospitalization | 4,208.4 (18.0) | 3,430.5 (17.9) | 445.8 (18.0) | 332.2 (18.4) |  |
| ≥2 Outpatient visits | 19,236.3 (82.0) | 15,733.0 (82.1) | 2,029.2 (82.0) | 1,474.1 (81.6) | 0.009 |
| **Atopy^a^** | 15,756.2 (67.2) | 12,914.8 (67.4) | 1,659.1 (67.0) | 1,182.3 (65.5) | 0.027 |
| **Asthma controller medication use** **in the year prior to the index visit** |  |  |  |  |  |
| ICS alone | 9,970.6 (42.5) | 8,146.9 (42.5) | 1,077.9 (43.6) | 745.9 (41.3) | 0.085 |
| ICS-LTRA | 602.2 (2.6) | 506.2 (2.6) | 64.1 (2.6) | 31.9 (1.8) |  |
| ICS-LABA | 254.2 (1.1) | 223.8 (1.2) | 19.9 (0.8) | 10.6 (0.6) |  |
| Other Controller^b^ | 259.6 (1.1) | 224.4 (1.2) | 24.4 (1.0) | 10.9 (0.6) |  |
| No Controller | 12,358.1 (52.7) | 10,062.3 (52.5) | 1,288.8 (52.1) | 1,007.1 (55.8) |  |
| **SABA doses per week 6 months prior to index visit** |  |  |  |  | 0.061 |
| 0 to 2 | 18,384.5 (78.4) | 15,074.5 (78.7) | 1,917.8 (77.5) | 1,392.1 (77.1) |  |
| >2 to < 4 | 798.4 (3.4) | 665.7 (3.5) | 70.9 (2.9) | 61.9 (3.4) |  |
| 4 to < 7 | 2,314.2 (9.9) | 1836.0 (9.6) | 266.6 (10.8) | 211.6 (11.7) |  |
| 7 or more | 1,947.7 (8.3) | 1587.2 (8.3) | 219.7 (8.9) | 140.7 (7.8) |  |
| **Number of exacerbations in the year prior to the index visit** |  |  |  |  |  |
| 0 | 20,555.8 (87.7) | 16,796.7 (87.6) | 2,172.9 (87.8) | 1,586.2 (87.8) |  |
| 1 | 1,505.3 (6.4) | 1,199.2 (6.3) | 184.3 (7.4) | 121.8 (6.7) | 0.060 |
| 2 | 646.7 (2.8) | 529.0 (2.8) | 63.3 (2.6) | 54.3 (3.0) |  |
| 3 or more | 737.0 (3.1) | 638.6 (3.3) | 54.5 (2.2) | 44.0 (2.4) |  |
| **Health care system access and continuity of care characteristics** |  |  |  |  |  |
| **Number of ambulatory care visits, ER visits, hospitalization for any cause in the year prior to the index visit, median (IQR)** |  |  |  |  |  |
| Ambulatory care visits | 4.0 (5.0) | 4.0 (5.0) | 4.0 (5.0) | 4.0 (5.0) | 0.047 |
| ER visits | 0.0 (1.0) | 0.0 (1.0) | 0.0 (1.0) | 0.0 (1.0) | 0.013 |
| Hospitalization | 0.0 (0.0) | 0.0 (0.0) | 0.0 (0.0) | 0.0 (0.0) | 0.046 |
| **Mode of birth** |  |  |  |  |  |
| Vaginal |  |  |  |  |  |
| Cesarian | 5,296.7 (22.6) | 4,358.7 (22.7) | 518.2 (20.9) | 419.8 (23.2) | 0.037 |
| Unknown |  |  |  |  |  |
| **Low birth weight (<2.5kg/5.5 lbs)** | 2,197.7 (9.4) | 1,825.0 (9.5) | 208.3 (8.4) | 164.3 (9.1) | 0.026 |
| **Maternal Characteristics** |  |  |  |  |  |
| **Maternal age at beginning of pregnancy, mean± SD** | 25.99 (5.64) | 26.02 (5.64) | 25.90 (5.64) | 25.86 (5.57) | 0.018 |
| **Maternal asthma control during pregnancy^c^** |  |  |  |  |  |
| Well Controlled | 1,565.2 (6.7) | 1,309.5 (6.8) | 151.9 (6.1) | 103.8 (5.7) |  |
| Partially controlled | 688.4 (2.9) | 588.6 (3.1) | 54.9 (2.2) | 44.9 (2.5) |  |
| Not controlled | 771.9 (3.3) | 641.8 (3.3) | 81.5 (3.3) | 48.6 (2.7) | 0.063 |
| Could not be addressed | 2,270.1 (9.7) | 1,876.7 (9.8) | 228.0 (9.2) | 165.4 (9.2) |  |
| No asthma | 18,149.2 (77.4) | 14,746.8 (77.0) | 1,958.7 (79.1) | 1,443.7 (79.9) |  |

Inhaled corticosteroids (ICS); Long-acting beta-2 agonists (LABA); Leukotriene receptor antagonists (LTRA); Short-acting beta-2 agonists (SABA).

a Atopy was defined using the following ICD-9/10 codes: 691, 692, 708, 477, 691.8, L20, L23, L50, J30.

b Other controller medication include: Theophylline alone, Long-acting beta-2 agonists alone, Leukotriene receptor antagonists alone.

c Maternal asthma defined as ≥ 1 hospitalization or ≥ 2 physician outpatient visits for asthma and measured in the 2 years before childbirth.

## **Figure S2. Antibiotic Treatment and Return for Infection Risk: Subgroup Effects in Children with Nonspecific Upper Respiratory Tract Infections**

The graph displays the Marginal Risk Ratio (mRR) with a 95% Confidence Interval (CI) comparing children exposed to oral antibiotics versus those unexposed, following a non-specific acute respiratory infection. The data is stratified into several subgroups, including age, sex, the type of doctor seen, asthma treatment status, and Short-acting beta-2 agonists (SABA) dose, with the total number of children noted for each subgroup.

## **Table S5. Sensitivity Analysis: Crude and Marginal Risk Differences and Ratios for Exacerbation over a 1-year Follow-Up Period Associated with Antibiotic Treatment Strategies in Children with Asthma Consulting for Nonspecific URTIs**

|  | **No. of first exacerbations/ No. of person-trial months** | **Crude incidence rate (95% CI) per 100 person-trial months** | **Crude RRs^a^**  **(95% CI)** | **Marginal RRs^b^**  **(95% CI)** | **Crude RD^a^**  **(95% CI)** | **Marginal RD^b^**  **(95% CI)** |
| --- | --- | --- | --- | --- | --- | --- |
| **All** | 1135/134292.1 | 0.85  (0.80; 0.90) | NA | N/A | NA | N/A |
| **No Antibiotic^c^** | 863/107251.1 | 0.80  (0.75; 0.86) | ref | ref | ref | ref |
| **Spectrum-Specific Effects** | | | | | | |
| **Narrow Spectrum** | 120/11761.9 | 1.02  (0.85; 1.22) | 1.28  (1.08; 1.49) | 1.31  (1.08; 1.49) | 0.22%  (0.04%; 0.40%) | 0.14%  0.05%; 0.24%) |
| **Broad Spectrum** | 152/15279.1 | 0.99  (0.84; 1.17) | 1.24  (1.08; 1.47) | 1.27  (1.08; 1.47) | 0.19%  (0.01%; 0.37%) | 0.10%  (0.03%; 0.18%) |
| **Class-Effects** | | | | | | |
| **Penicillins** | 107/11621.1 | 0.92  (0.75; 1.11) | 1.14  (0.94; 1.40) | 1.18  (1.02; 1.37) | 0.12%  (-0.07%; 0.30%) | 0.08%  (0.02%; 0.15%) |
| **Cephalosporins** | 45/3843.2 | 1.17  (0.85; 1.57) | 1.46  (1.08; 1.96) | 1.49  (1.13; 1.77) | 0.37%  (0.02%; 0.71%) | 0.21%  (0.04%; 0.32%) |
| **Macrolides** | 99/9970.4 | 0.99  (0.81; 1.21) | 1.23  (1.00; 1.52) | 1.17  (1.01; 1.35) | 0.19%  (-0.01%; 0.39%) | 0.08%  (0.01%; 0.14%) |
| **Other types** | 21/1606.3 | 1.31  (0.81; 2.00) | 1.62  (1.06; 2.50) | 1.68  (1.05; 2.21) | 0.50%  (-0.06%; 1.06%) | 0.27%  (0.07%; 0.48%) |

^a^Crude effects are unadjusted for confounders

^b^Marginal effects are adjusted for confounders via inverse probability of treatment weighting. Confounders included in the propensity score model used to estimate weights include: Sex, Age, Rurality, Social assistance, Location of index visit, Type of infection at index visit, Type of physician at index visit, Asthma diagnosis criteria, Year of asthma diagnosis, Month of CE, Atopy, Asthma controller medication, Number of asthma exacerbation in the previous year, Number of health care visits for any cause at outpatient clinic in the previous year, Number of health care visits for any cause at ED in the previous year, Number of hospitalizations in the previous year, Low birth weight, Mode of delivery, Maternal age at the beginning of pregnancy, Asthma control during pregnancy

^c^Reference category

## **Table S6. Sensitivity Analysis: Crude and Marginal Risk Differences and Ratios for Exacerbation associated with Antibiotic Treatment Strategies in Children with Newly Diagnosed Asthma consulting for Nonspecific URTIs**

|  | **No. offirst exacerbations/ No. of person-trial months** | **Crude incidence rate (95% CI) per 100 person-trial months** | **Crude RRs^a^**  **(95% CI)** | **Marginal RRs^b^**  **(95% CI)** | **Crude RD^a^**  **(95% CI)** | **Marginal RD^b^**  **(95% CI)** |  |
| --- | --- | --- | --- | --- | --- | --- | --- |
| **All** | 151/19855.1 | 0.76  (0.64; 0.88) | NA | N/A | NA | N/A |  |
| **No Antibiotic^c^** | 104/14172.2 | 0.73  (0.59; 0.87) | ref | ref | ref | ref |  |
| **Spectrum-Specific Effects** | | | | | | | |
| **Narrow Spectrum** | 18/2309.1 | 0.78  (0.42; 1.14) | 1.06  (0.64; 1.75) | 1.27  (0.74; 1.83) | 0.05%  (−1.34%; 1.45%) | 0.01%  (-0.15%; 0.33%) |  |
| **Broad Spectrum** | 29/3373.8 | 0.86  (0.55; 1.17) | 1.17  (0.78; 1.77) | 1.31  (0.80; 1.78) | 0.13%  (−0.58%; 0.83%) | 0.12%  (-0.06%; 0.29%) |  |
| **Class-Effects** | | | | | | | |
| **Cephalosporins** | 7/703.9 | 0.99  (0.40; 2.05) | 1.36  (0.63; 2.91) | 1.39  (0.57; 2.16) | 0.26%  (−0.49%; 1.01%) | 0.14%  (-0.16%; 0.43%) |  |
| **Macrolides** | 12/1419.2 | 0.85  (0.44; 1.48) | 1.15  (0.63; 2.09) | 1.05  (0.57; 1.52) | 0.11%  (−0.39%; 0.61%) | 0.01%  (-0.17%; 0.21%) |  |
| **Penicillins** | 13/1768.9 | 0.73  (0.39; 1.26) | 1.00  (0.56; 1.78) | 1.03  (0.66; 1.47) | 0.01%  (−0.42%; 0.42%) | 0.00%  (-0.18%; 0.17%) |  |
| **Other types** | 5/299.9 | 1.67  (0.54; 3.89) | 2.27  (0.93; 5.57) | 2.43  (0.61; 4.04) | 0.93%  (−0.53%; 2.40%) | 0.58%  (-0.22%; 1.23%) |  |

^a^Crude effects are unadjusted for confounders

^b^Marginal effects are adjusted for confounders via inverse probability of treatment weighting. Confounders included in the propensity score model used to estimate weights include: Sex, Age, Rurality, Social assistance, Location of index visit, type of physician at index visit, asthma diagnosis criteria, Year of asthma diagnosis, Month of the index visit, Atopy, Asthma controller medication, Average weekly dose of short-acting beta-2 agonist (SABA), number of asthma exacerbation in the previous year, Number of health care visits for any cause at outpatient clinic in the previous year, Low birth weight, Preterm birth, Mode of delivery, Maternal age at the beginning of pregnancy, Maternal asthma status, and Asthma control during pregnancy

^c^Reference category

## **Table S7. Sensitivity Analysis: Crude and Marginal Risk Differences and Ratios for Exacerbation Associated with Antibiotic Exposure Excluding Children with an Exacerbation Between the Index Visit and the Start of Follow-up**

|  | **No. of first exacerbations/ No. of person-trial months** | **Crude incidence rate (95% CI) per 100 person-trial months** | **Crude RRs^a^**  **(95% CI)** | **Marginal RRs^b^**  **(95% CI)** | **Crude RD^a^**  **(95% CI)** | **Marginal RD^b^**  **(95% CI)** |
| --- | --- | --- | --- | --- | --- | --- |
| **All** | 614/131537.1 | 0.47  (0.43; 0.51) | NA | N/A | NA | N/A |
| **No Antibiotic^c^** | 480/105313.6 | 0.46  (0.42; 0.50) | ref | ref | ref | ref |
| **Spectrum-Specific Effects** | | | | | | |
| **Narrow Spectrum** | 61/11577.4 | 0.52  (0.40; 0.68) | 1.16  (0.88; 1.53) | 1.24  (0.99; 1.52) | 0.07%  (−0.13%; 0.27%) | 0.06%  (0.01%; 0.14%) |
| **Broad Spectrum** | 73/14646.1 | 0.49  (0.39; 0.63) | 1.09  (0.85; 1.39) | 1.20  (0.91; 1.41) | 0.04%  (−0.14%; 0.23%) | 0.04% (-0.03%; 0.10%) |
| **Effects of Antibiotic Classes Compared to No Antibiotic** | | | | | | |
| **Penicillins** | 51/11407.0 | 0.45  (0.33; 0.59) | 0.98  (0.74; 1.31) | 0.97  (0.76; 1.20) | -0.01%  (-0.14%; 0.12%) | -0.01%  (-0.06%; 0.05%) |
| **Cephalosporins** | 19/3758.1 | 0.51  (0.30; 0.79) | 1.11  (0.70; 1.75) | 1.13  (0.70; 1.53) | 0.05%  (-0.18%; 0.28%) | 0.03%  (-0.08%; 0.13%) |
| **Macrolides** | 51/9518.0 | 0.54  (0.40; 0.70) | 1.18  (0.88; 1.57) | 1.10  (0.82; 1.38) | 0.08%  (-0.07%; 0.23%) | 0.03%  (-0.05%; 0.10%) |
| **Other types** | 13/1540.4 | 0.84  (0.45; 1.44) | 1.85  (1.07; 3.20) | 1.81  (0.94; 2.64) | 0.39%  (-0.07%; 0.85%) | 0.20%  (0.00%; 0.37%) |

^a^Crude effects are unadjusted for confounders

^b^Marginal effects are adjusted for confounders via inverse probability of treatment weighting. Confounders included in the propensity score model used to estimate weights include: Sex, Age, Rurality, Social assistance, Location of index visit, type of physician at index visit, asthma diagnosis criteria, Year of asthma diagnosis, Month of the index visit, Atopy, Asthma controller medication, Average weekly dose of short-acting beta-2 agonist (SABA), number of asthma exacerbation in the previous year, Number of health care visits for any cause at outpatient clinic in the previous year, Low birth weight, Preterm birth, Mode of delivery, Maternal age at the beginning of pregnancy, Maternal asthma status, and Asthma control during pregnancy

^c^Reference category

## **Table S8. E-Value Calculation for Overall Risk Ratios of Exacerbation for Each Antibiotic Treatment Strategy**

| **Exposure** | **Summary mRR (95% CI)** | **E-value (LCI)*** | **Interpretation** |
| --- | --- | --- | --- |
| **Spectrum-Specific Effects** | | | |
| **Narrow Spectrum** | 1.26 (0.99 – 1.53) | 1.97 (1.00) | Average estimate can be explained away with weak unmeasured confounding (RR_EU_=1.97 & RR_UY_=1.97) ** |
| **Broad Spectrum** | 1.28 (1.03 – 1.54) | 2.02 (1.37) | Average estimate can be explained away with moderate unmeasured confounding (RR_EU_=2.02 & RR_UY_=2.02) |
| **Class-Specific Effects** | | | |
| **Penicillins** | 1.01 (0.83 – 1.22) | 1.13 (1.00) | Not robust — CIs cross 1; effects could be explained by mild–moderate confounding |
| **Cephalosporins** | 1.16 (0.78 – 1.56) | 1.62 (1.00) | Not robust — CIs cross 1; effects could be explained by mild–moderate confounding |
| **Macrolides** | 1.24 (0.92 – 1.55) | 1.93 (1.00) | Not robust — CIs cross 1; effects could be explained by mild–moderate confounding |
| **Other antibiotics** | 1.69 (0.86 – 2.50) | 2.73 (1.00) | Not robust — CIs cross 1; effects could be explained by mild–moderate confounding |

* LCI refers to the E-value for the lower bound of the 95% confidence interval. It represents the minimum strength of association that an unmeasured confounder would need to have with both the exposure and the outcome (conditional on measured covariates) to fully explain away the observed association.

** RR_EU_ and RR_UY_ denote the minimum required risk ratios relating the unmeasured confounder to the exposure (RR_EU_) and to the outcome (RR_UY_). These correspond to the same confounding strength encoded in the E-value and represent the magnitude of bias an unmeasured confounder would need to induce to account for the observed effect estimate.
